# Oral Administration of *Latilactobacillus sakei* ADM14 Improves Lipid Metabolism and Fecal Microbiota Profile Associated With Metabolic Dysfunction in a High-Fat Diet Mouse Model

**DOI:** 10.3389/fmicb.2021.746601

**Published:** 2021-10-06

**Authors:** Sung-Min Won, Min Ju Seo, Min Ju Kwon, Kye Won Park, Jung-Hoon Yoon

**Affiliations:** Department of Food Science and Biotechnology, Sungkyunkwan University, Suwon, South Korea

**Keywords:** *Latilactobacillus sakei*, probiotics, obesity, lipid metabolism, microbiome

## Abstract

Effects of *Latilactobacillus sakei* ADM14 on changes in lipid metabolism and fecal microbiota composition were studied in high-fat diet (HFD) mouse model. The mice were divided into three groups: normal diet (ND), high-fat diet (HD), and HFD plus *L. sakei* ADM14 (HDA). Oral administration of *L. sakei* ADM14 daily for 10weeks decreased body weight gain, fat tissue mass, and liver weight in mice and reduced the size of histologically stained liver adipocytes. In addition, serum total cholesterol, triglycerides, and blood glucose decreased significantly. *Latilactobacillus sakei* ADM14 regulated the expression of genes related to lipid metabolism in epididymal adipose tissue and liver and induced changes in the composition of fecal microbiota, thereby improving energy harvests and changing metabolic disorder-related taxa. A significant decrease (*p*<0.05) in the *Firmicutes* to *Bacteroidetes* ratio was found in the HDA group compared to the HD group, particularly due to the difference in the relative abundance of the *Bacteroidetes* between the two groups over 10weeks. Differences in proportions of some taxa reported to have correlation with obesity were also found between HD and HDA groups. These results suggest that *L. sakei* ADM14 can have a positive effect on metabolic disorders such as obesity and fatty liver through effective regulation of host lipid metabolism and gut microbiota.

## Introduction

Obesity has emerged as an important health problem through a rapid increase in incidence worldwide. Excessive body weight gain and body fat accumulation pose a serious threat, leading to the onset of obesity and metabolic disorders such as diabetes and cardiovascular disease (Bastien et al., 2014). A high-fat diet (HFD) affects elevated serum cholesterol and triglycerides (TG) and increases the risk of chronic low-grade inflammation from obesity, along with adipose tissue enlargement (Dewulf et al., 2011). The influence of metabolic disorders in combination with the HFD can also damage the liver. Recently, the association and risk of obesity with nonalcoholic fatty liver disease (NAFLD) has received a lot of attention. NAFLD includes a variety of symptoms ranging from simple steatosis to nonalcoholic fatty hepatitis, cirrhosis, or hepatocellular carcinoma (Wong et al., 2015), and more than 80% of obese individuals in western countries are affected by NAFLD (Younossi, 2019). Recently, several studies have focused on gut microbiota as a new environmental factor contributing to the relationship between obesity and NAFLD (Le Roy et al., 2013; Tilg et al., 2016; Da Silva et al., 2018). The gut microbial community consists of thousands of bacterial species, coexisting with the host, and has a significant influence on the physiology and metabolism of the host (Turnbaugh et al., 2006; Ridaura et al., 2013). Recently, a link between the effects of choline deficiency on fatty liver development and changes in the human gut microbiota was confirmed (Spencer et al., 2012), and it was revealed that changes in the gut microbiota regulate the progression of NAFLD (Buzzetti et al., 2016). Thus, the regulation of gut microbial communities may suggest new therapeutic strategies in the management of metabolic diseases such as obesity and NAFLD. Probiotics regulate the gut microbiome and have proven beneficial effects on metabolic symptoms. They are also known to be effective in improving lipid profiles and hyperlipidemia, while affecting lipid metabolism (Roller et al., 2004; Sun and Buys, 2015).

In our previous study, a lactic acid bacterium, designated ADM14, was found to have anti-adipogenic effect by reducing significantly intracellular triglyceride content on 3T3-L1 adipocytes and by decreasing the expression of five adipogenic marker genes (Won et al., 2020a). The strain ADM14 was identified as a member of *Lactobacillus sakei* in the study of Won et al. (2020a), but *L. sakei* has been recently reclassified as *L. sakei* (Zheng et al., 2020). The aim of this study was to investigate the effect of *L. sakei* ADM14 on host lipid metabolism, fatty liver, and metabolic problems in HFD mouse model. In addition, changes in gut microbiota structure on the host were analyzed through fecal microbiome analysis the relationship between the gut-liver axis was evaluated.

## Materials and Methods

### Bacterial Strain Preparation and Growth Conditions

*Latilactobacillus sakei* (*L. sakei*) ADM14 was cultured using De Man-Rogosa-Sharpe (MRS, BD Difco, Sparks, MD, United States) agar at 30°C for 24h and was stored at −80°C in 20% glycerol (v/v; Georgiachem, GA, United States) for cryopreservation until it was used in experiments.

### Animals, Diets, and Experimental Design

The animal care and studies were conducted in accordance with the Animal Care and Use Committee of the College of Biotechnology at Sungkyunkwan University (approval date: 07-09-2019, approval number: SKKUIACUC-18-04-14-3). Male C57BL/6J mice aged 5weeks were purchased from RaonBio Inc. (Yongin, Republic of Korea) and maintained under controlled temperature and humidity (24±2°C, 50±10%) with a 12h light/dark cycle. After a 1-week acclimation period, 6-week-old mice were randomly divided into three groups (*n*=8): normal diet (ND), high-fat diet (HD), and high-fat diet plus *L. sakei* ADM14 (HDA, 10^8^–10^9^CFU per 200μl 0.85% saline). The ND group received a normal chow diet (10% of energy from fat, 16.1kJ, RaonBio Inc.) for 10weeks and the HD group received a HFD (60% of energy from fat, 21.9kJ, RaonBio Inc.) for 10weeks (Supplementary Table S1). The HDA group received a HFD for 10weeks and *L. sakei* ADM14 by daily oral administration. Food and water were fed *ad libitum*. Live *L. sakei* ADM14 was administered by oral gavage at a concentration of 10^8^–10^9^CFU per 200μl 0.85% saline daily as recommended by the WHO and the Korea Food and Drug Administration. During the experiment, food intake and body weight were measured weekly. Fecal samples were collected weekly and stored at −80°C. The food efficiency ratio (FER) was expressed as total body weight gained from the diet divided by total diet consumed during the animal experiments, and total calorie intake was calculated as the total amount of diet consumed during the animal experiment multiplied by the caloric value of the diet. The mice that died during the experiment of 10weeks were excluded, and the mice with the maximum and minimum values in weight were also excluded. Finally, 4–6 mice per group were used in further analyses. At the end of the experiment, mice were fasted for 16h and sacrificed under anesthesia. After sacrifice, visceral organs (liver, spleen, and kidney) and epididymal and subcutaneous fat pad were collected and weighed. Epididymal fat pads and liver were preserved by freezing in liquid nitrogen for genetic analysis. A portion of the liver was frozen and stained with Oil Red O (Sigma-Aldrich, St. Louis, MO, United States) for histological study. Blood was collected *via* cardiac puncture and was centrifuged for 10min at 3,000rpm for serum separation.

### Serum Analysis

Alanine transaminase (ALT), aspartate transaminase (AST), total cholesterol, glucose, triglyceride (TG), high-density lipoprotein (HDL), and low-density lipoprotein (LDL) levels were measured using a biochemical automatic analyzer (AU480, Beckman Coulter Inc., Brea, CA, United States) according to the manufacturer’s instructions.

### RNA Extraction and Quantitative Real-Time PCR

Total RNA was extracted from epididymal fat tissue and liver using an RNeasy Mini Kit (Qiagen, Hilden, Germany) and TRIzol (Invitrogen, Carlsbad, CA, United States) according to the manufacturer’s protocol. First-strand complementary DNA was synthesized using a Veriti™ 96-Well Thermal Cycler machine (Thermo Scientific, Waltham, MA, United States) by mixing the extracted total RNA and ReverTra Ace Master Mix (Toyobo, Osaka, Japan). A mixture of Power SYBR Premix ExTaq (RP041A; Takara, Shiga, Japan), primers and cDNA was used for amplification using a thermal cycler machine (Takara). Gene expression was normalized by a housekeeping gene, *36B4*. The primer sequences for the genes are shown in Supplementary Table S2.

### 16S rRNA Gene Sequence Analysis of Gut Microbiota and Bioinformatics

For microbiome analysis, total genomic DNA was extracted from fecal samples using a QIAamp DNA Stool Mini Kit (Qiagen) according to the manufacturer’s protocol. The first amplification from the total genomic DNA was performed in the V3 to V4 regions with primer sequences of 16S rRNA gene as shown in Supplementary Table S3 (Fadrosh et al., 2014). A second amplification was performed by attaching an Illumina NexTera barcode to the first amplification product. Sequencing was performed according to the method of Chunlab Inc. (Seoul, Republic of Korea) using an Illumina MiSeq sequencing system (Illumina, San Diego, CA, United States). Taxonomic profiling and sequencing data analysis were performed using the Illumina platform (Chunlab Inc.). Alpha diversity was calculated using OTU information and expressed as the Chao 1 and Shannon index. The microbiota structure between groups was measured to principal coordinate analysis (PCoA) at the genus level using the beta diversity index. The linear discriminant analysis effect size (LEfSe) method was performed using latent Dirichlet allocation (LDA) score 3.0 cutoff and value of *p*<0.05.

### Statistical Analysis

Statistical analyses were conducted using SPSS ver. 19.0 (SPSS Inc., Chicago, IL, United States). Data are presented as mean±SEM. Significant differences in the gene expression of tissue between animal experimental groups were determined by unpaired Student’s *t*-test. For relative abundance analysis of the gut microbiome, significant differences between groups were determined using the Wilcoxon rank-sum test. Values were considered statistically significant when *p*<0.05.

## Results

### Effects of *L. sakei* ADM14 on Body, Liver, and Fat Tissue Weight

**Figure 1:**
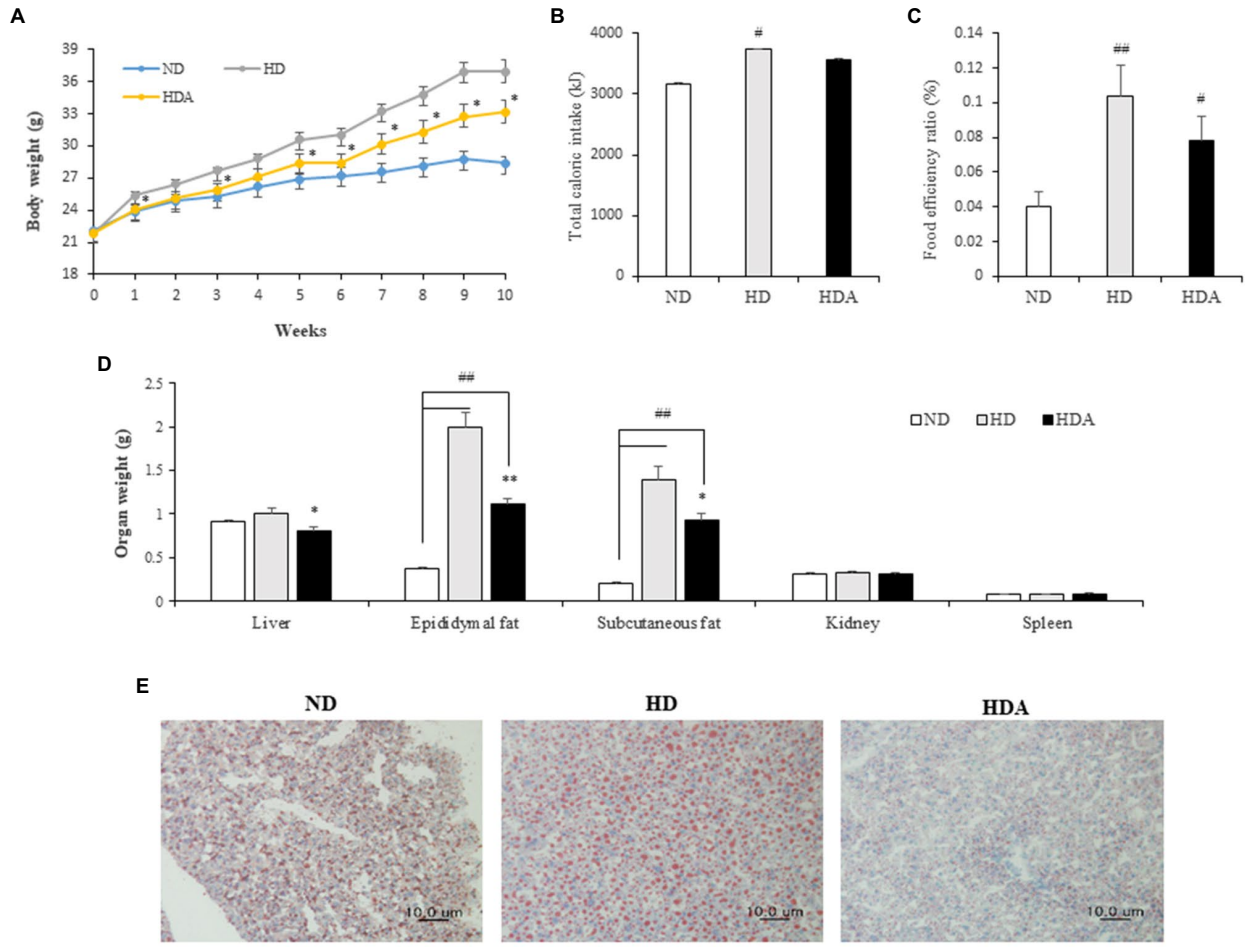
Effect of administering *Lactobacillus sakei* ADM14 on body indicators in high-fat diet (HFD) mouse model. **(A)** Weight change of mice groups. **(B)** Total caloric intake for 10weeks. **(C)** Food efficiency ratio (FER) for 10weeks. **(D)** Organs weights of mice after sacrifice. **(E)** Histological analysis of liver stained with Oil Red O. ND, normal diet group; HD, high-fat diet group; and HDA, high-fat diet plus *L. sakei* ADM14 group. The results are shown as mean±SEM (*n*=5–7). Significant differences between HD and ND are indicated as ^#^*p*<0.05 and ^##^*p*<0.01. Significant differences between HDA and ND are indicated as ^#^*p*<0.05 and ^##^*p*<0.01. Significant differences between HD and HDA are indicated as ^*^p<0.05 and ^**^p<0.01.

Changes in weight between the groups of mice were observed for 10weeks. The weight of group fed the HFD increased more rapidly (Figure 1A). After 10weeks, the average weight gains in the ND, HD, and HDA groups were 6.31±0.56, 15.03±1.25, and 11.30±1.01g, respectively. Compared with the HD group, the weight gain of the HDA group decreased significantly by 24.7% (*p*<0.05). While the total caloric intake was significantly different (ND vs. HD, *p*<0.01; ND vs. HDA, *p*<0.05) between the ND and HFD intake groups, there was no significant difference between the HD and HDA groups (Figure 1B). The FER increased significantly (*p*<0.01) in HD compared to ND, but there was no significant difference despite a 12.9% decrease in HDA compared to HD (Figure 1C). No significant weight changes were observed in the kidneys and spleen, of each group (Figure 1D). There was a significant increase (*p*<0.01) in the mass of epididymal fat and subcutaneous fat in HD compared to ND (Figure 1D). In contrast, there was a significant decrease of 44.5% in epididymal fat (*p*<0.01) and 33.8% in subcutaneous fat (*p*<0.05) in HDA compared to HD (Figure 1D). The liver weight was not significantly different between ND and HD, but a significant decrease (*p*<0.05) was observed in HDA compared to HD (Figure 1D). In the histological analysis of the liver using Oil Red O staining, it was observed that the size of stained adipocytes decreased in HDA compared to HD (Figure 1E).

### Effects of *L. sakei* ADM14 on Serum Biochemical Parameters

The concentrations of total cholesterol, glucose, TG, and HDL of the serum in the HD group were significantly (*p*<0.01) higher than those of the ND group (Table 1). Compared with the HD group, total cholesterol, glucose, and TG of the serum decreased in the HDA group administered with *L. sakei* ADM14 (Table 1). Total cholesterol decreased by 17.0% (*p*<0.05), glucose decreased by 29.9% (p<0.05), and TG decreased by 34.2% (*p*<0.01). Compared to the HD group, the HDA group showed a significant increase (p<0.05) in HDL, and the LDL decreased by 9.0% but there was no significant difference between them. ALT and AST were analyzed and there was no significant difference among the groups (Supplementary Figure S1).

**Table 1:** Biochemical parameter of serum.

	ND	HD	HDA
Total cholesterol	105.00±3.67	185.25±11.12^##^	153.75±6.41^*^
Glucose	70.75±3.33	162.75±7.39^##^	114.00±15.64^*^
TG	61.00±1.87	86.25±4.96^##^	56.75±2.53^**^
HDL	83.25±1.89	101.25±1.89^##^	110.25±3.09^*^
LDL	21.75±1.44	24.75±0.75	22.50±0.87

Values are shown as the means±SEM (*n*=4–5). Significant differences between HD and ND are indicated as follow:

## *p*<0.01.

Significant differences between HD and HDA are indicated as follow:

* *p*<0.05;

** *p*<0.01.

### Effects of *L. sakei* ADM14 on Genes of Lipid Metabolism in Epididymal Fat Tissue and Liver

**Figure 2:**
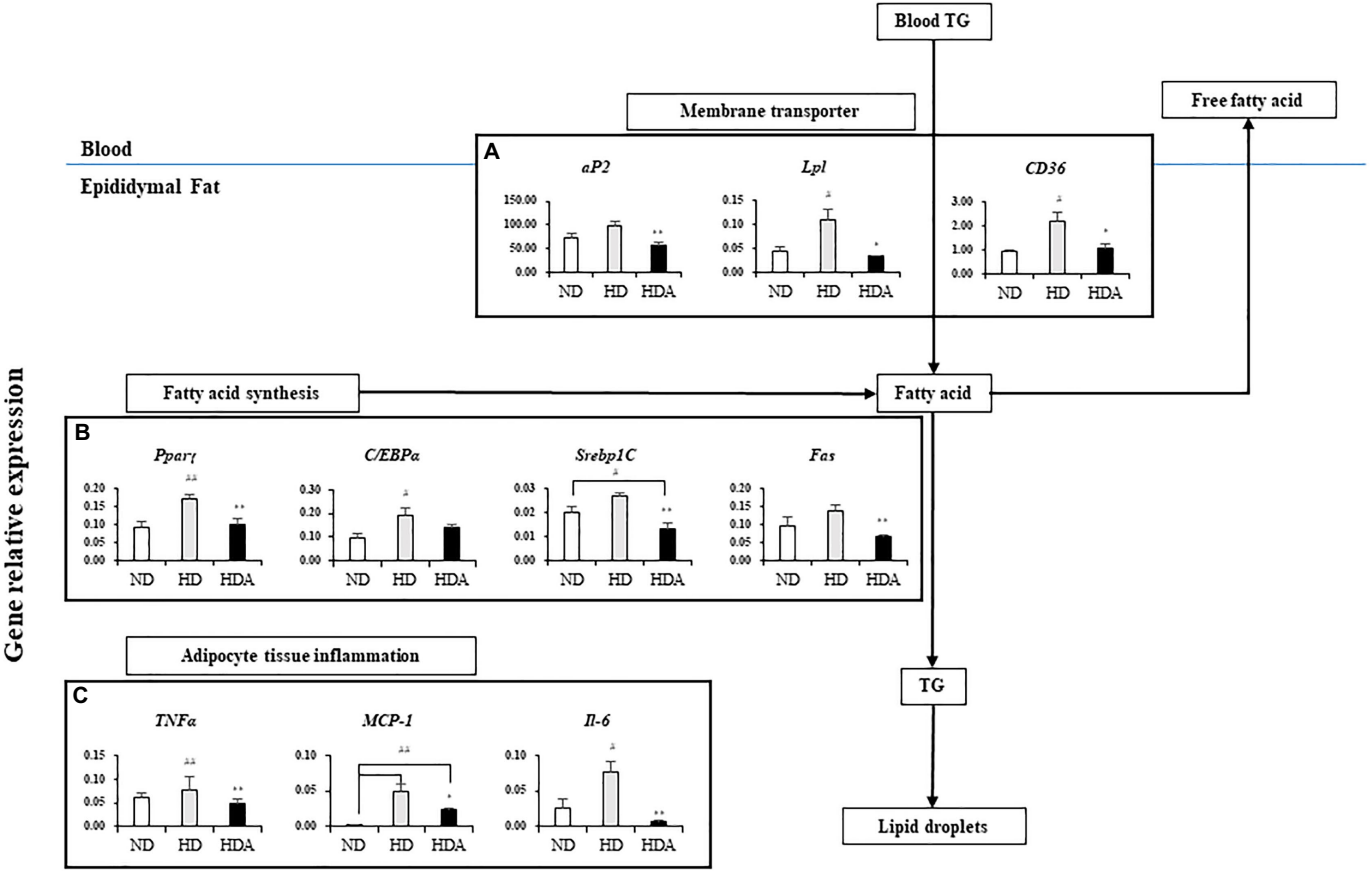
Effects of administering *L. sakei* ADM14 on the transcription of genes related to lipid metabolism in epididymal fat tissue. The results are shown as mean±SEM (*n*=5). Significant differences between HD and ND are indicated as ^#^*p*<0.05 and ^##^*p*<0.01. Significant differences between HDA and ND are indicated as ^#^*p*<0.05 and ^##^*p*<0.01. Significant differences between HD and HDA are indicated as ^*^p<0.05 and ^**^p<0.01.

The results for the mRNA expression of genes for lipid metabolism in epididymal fat tissue are shown in Figure 2. The expressions of genes related to lipid metabolism in epididymal fat tissue of HD group increased when compared to the ND group (Figure 2). Compared to the HD group, the HDA group administered with *L. sakei* ADM14 significantly downregulated (*p*<0.01) peroxisome proliferator-activated receptor gamma (*Pparγ*), sterol regulatory element-binding protein-1C (*Srebp1C*), and fatty acid synthetase (*Fas*), of four genes related to fatty acid synthesis (Figure 2B). In addition, the expression of adipocyte protein 2 (*aP2*) was also significantly decreased (*p*<0.01) in the HDA compared to the HD (Figure 2A). Likewise, the expressions of *Pparγ*-activated genes, lipoprotein-lipase (*Lpl*), and cluster of differentiation 36 (*CD36*) were significantly decreased (*p*<0.05) in the HDA compared to the HD (Figure 2A). The expressions of tumor necrosis factor-alpha (*TNFα*; *p*<0.01), interleukin 6 (*IL-6*; *p*<0.01), and monocyte chemoattractant protein-1 (*MCP-1*; *p*<0.05) related to pro-inflammatory cytokines in the HDA were significantly decreased compared to the HD group (Figure 2C).

**Figure 3:**
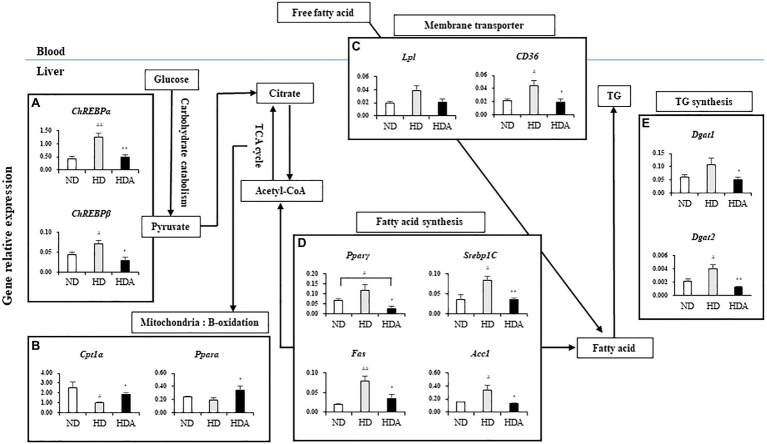
Effects of administered *L. sakei* ADM14 on the transcription of genes related to glucose and lipid metabolism in the liver. The results are shown as mean±SEM (*n*=5). Significant differences between HD and ND are indicated as ^#^*p*<0.05 and ^##^*p*<0.01. Significant differences between HDA and ND are indicated as ^#^*p*<0.05 and ^##^*p*<0.01. Significant differences between HD and HDA are indicated as ^*^p<0.05 and ^**^p<0.01.

To investigate the effect of *L. sakei* ADM14 on lipid metabolism in the liver, expression of overall related genes was investigated (Figure 3). There were also differences in the expressions of genes related to lipid metabolism in the liver between ND and HDA groups (Figure 3). Compared with HD, the lipid production-related genes, *Pparγ* (*p*<0.05), *Srebp1C* (*p*<0.01), *Fas* (*p*<0.05), and acetyl-CoA carboxylase 1 (*Acc1*; *p*<0.05), were significantly decreased in HDA (Figure 3D). In addition, the significantly increased expressions (*p*<0.05) of β-oxidation-related genes, carnitine palmitoyltransferase1 alpha (*Cpt1α*) and peroxisome proliferator-activated receptor alpha (*Pparα*), were found in HDA (Figure 3B). Carbohydrate-response element-binding proteins, *ChREBPα* (*p*<0.01) and *ChREBPβ* (*p*<0.05), which are major transcriptional regulatory factors that are activated by glucose catabolism in the liver and regulates lipogenesis, were observed to be significantly downregulated in HDA (Figure 3A). There was no significant change in the level of *Lpl* expression, but *CD36* expression showed a significant decrease (*p*<0.05) in HDA (Figure 3C). As a result of observing the expression of diglyceride acyltransferase (*Dgat*) that catalyzes TG formation, it was found that both *Dgat1* (*p*<0.05) and *Dgat2* (*p*<0.01) were significantly decreased (Figure 3E).

### Effects of *L. sakei* ADM14 on Changes in Ratio and Composition of Fecal Microbiota

On analyzing alpha diversity, there was no significant difference in the Chao1 index of each group, but there was a significant increase (*p*<0.05) in the HDA group in the Shannon index compared to the ND group (Supplementary Figure S2). On the other hand, there was no significant change between the HD group and the HDA group.

**Figure 4:**
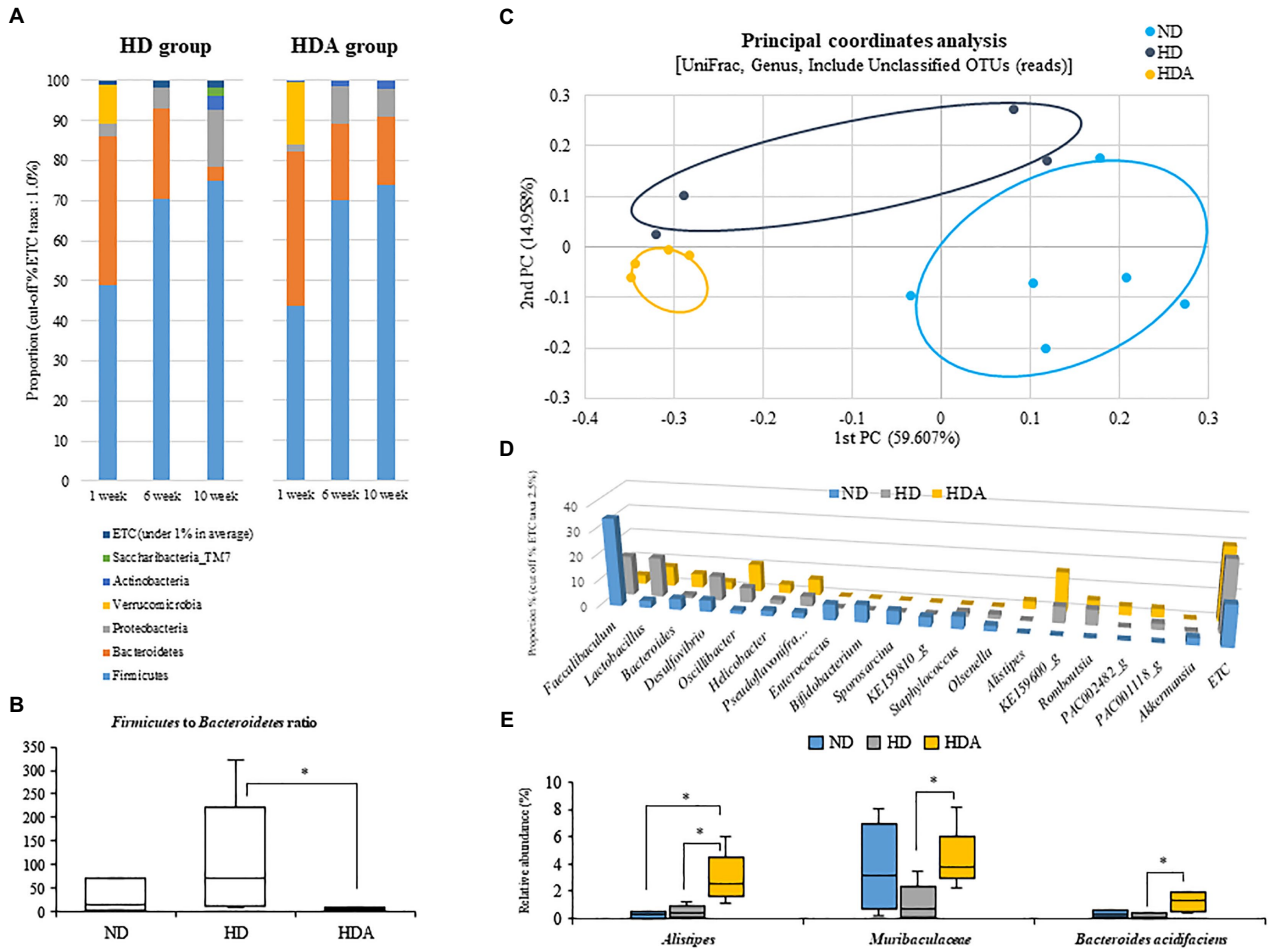
Effect of administered *L sakei* ADM14 on fecal microbiome composition. **(A)** Changes in fecal microbiota in HD and HDA groups at the phylum level in 1, 6, and 10weeks. **(B)** *Firmicutes* to *Bacteroidetes* ratios between groups at 10weeks. **(C)** Fecal microbiota composition of groups at 10weeks is shown by the genus level UniFrac principal coordinates analysis (PCoA). **(D)** Main fecal microbiota composition of genus level (cut off taxa 2.5%) at 10weeks. **(E)** The relative abundance of specific taxa in fecal microbiota at 10weeks. The results are shown as mean±SEM (*n*=4–6). Significant differences between groups are indicated as ^*^p<0.05.

Changes of fecal microbiota in the phylum level were assessed at 1, 6, and 10weeks using each group (Figure 4A). An increase in *Firmicutes* and a decrease in *Bacteroidetes* from 1 to 10weeks were observed in both HD and HDA groups, but each showed a difference in the range of variation. The *Firmicutes* ratios at 1, 6, and 10weeks were 49.02, 70.55, and 74.98%, respectively, in the HD group and 43.58, 70.02, and 74.04%, respectively, in the HDA group. There was no significant difference in the *Firmicutes* ratio between the two groups at 6 and 10weeks. The *Bacteroidetes* ratios at 1, 6, and 10weeks were 37.14, 22.37, and 3.56%, respectively, in the HD group and 38.49, 19.20, and 16.72%, respectively, in the HDA group. At 10weeks, the *Bacteroidetes* ratios of the two groups showed a significant difference (*p*<0.05). In addition, the *Proteobacteria* ratios at 1, 6, and 10weeks were 2.95, 5.28, and 14.16%, respectively, in the HD group and 2.01, 9.50, and 7.21% at 1, 6, and 10weeks, respectively, in the HDA group. The HD group showed a steady increase in the *Proteobacteria* ratios, but the HDA group decreased again from 6 to 10weeks in the *Proteobacteria* ratios. The proportions of the three major phyla, *Firmicutes*, *Bacteroidetes*, and *Proteobacteria*, at 10weeks of HD and HDA groups were similar with those of the two groups shown in cecal microbiota from our previous study (Won et al., 2020b), even though the proportion of the *Proteobacteria* in HDA group is higher than that of *Bacteroidetes* in the study of Won et al. (2020b). However, the *Verrucomicrobia*, which was the second major phylum in the cecal microbiota of HD group, was found only as minor phylum at 6 and 10weeks in fecal microbiota of this study (Won et al., 2020b; Figure 4A).

**Figure 5:**
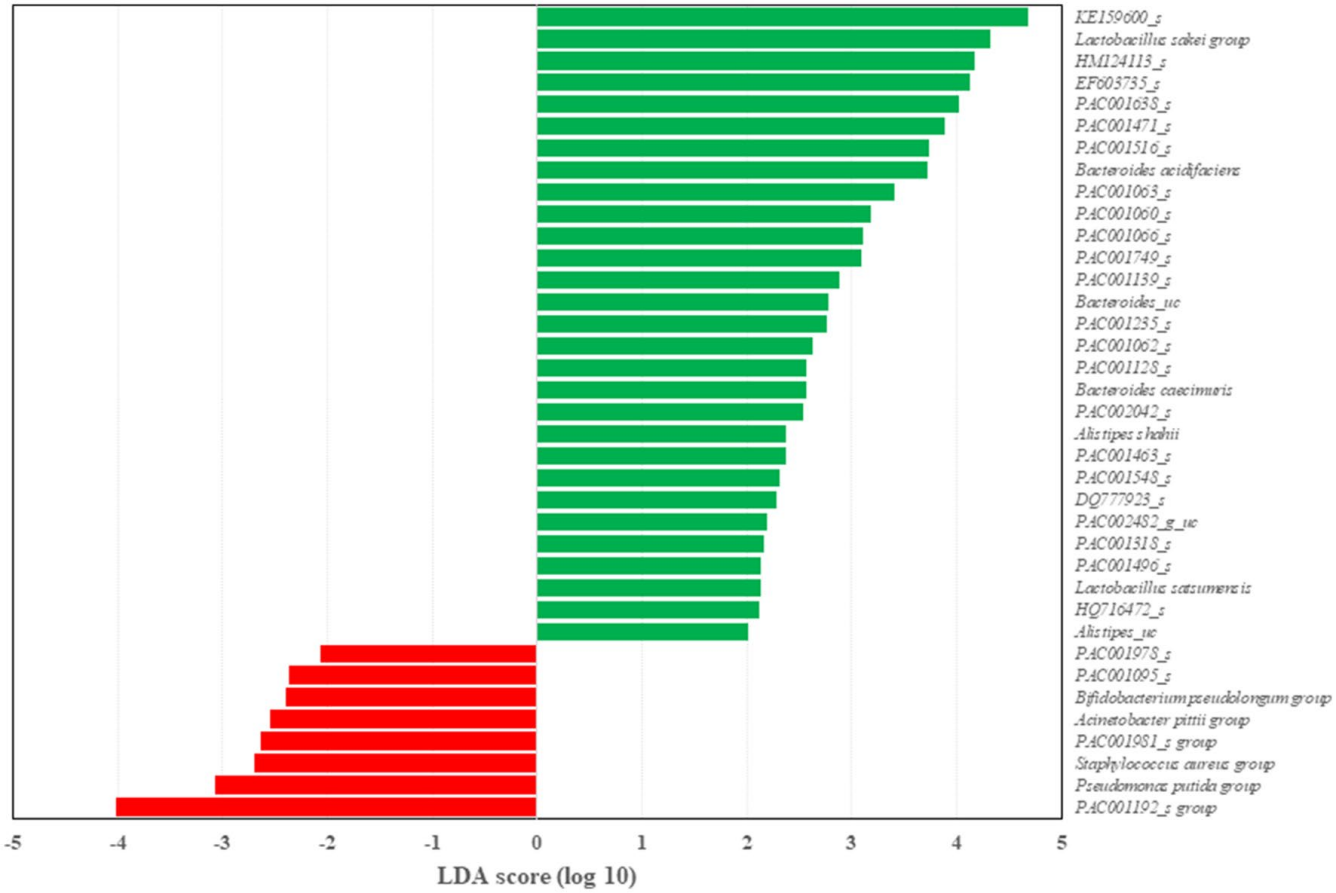
Differentially represented genus between HD and HDA group through linear discriminant analysis effect size (LEfSe) analysis. Latent Dirichlet allocation (LDA) score indicates the effect size. *n*=4–6 per group.

Two dominant phyla, *Firmicutes* and *Bacteroidetes*, were compared between the three groups at 10weeks (Figure 4B). The *Firmicutes* to *Bacteroidetes* ratio was significantly decreased (*p*<0.05) in the HDA group compared to the HD group as well as ND group. At the genus level, UniFrac PCoA showed a tendency to separate among the three groups (Figure 4C). The percentage of major genus composition in the ND, HD, and HDA groups were investigated (Figure 4D). The proportions of genera *Bacteroides*, *Oscillibacter*, *Helicobacter*, *Pseudoflavonifractor*, *Alistipes*, KE159600_g, PAC002482_g, and PAC001118_g were higher in the HDA group than in other two groups. On the other hand, the proportions of genera *Faecalibaculum*, *Lactobacillus*, *Desulfovibrio*, *Staphylococcus*, *Olsenella*, and *Romboutsia* were lower in the HDA group compared to the HD group. Significant increases (*p*<0.05) in levels of family *Muribaculaceae* and *Bacteroides acidifaciens* were found between the HDA and HD groups (Figure 4E). Significant increase (*p*<0.05) in genus *Alistipes* was found in the HDA group compared to the ND and HD groups (Figure 4E). LEfSe analysis was calculated to identify specific bacterial taxa dominant in the HD and HDA groups (Figure 5). *Pseudomonas* and PAC000677_g were the core genus microbiota of the HD group, and *Bacteroides*, PAC002482_g, *Alistipes*, PAC001063_g, PAC000198_g, and PAC001066_g were identified as the dominant genus microbiota of the HDA group.

## Discussion

The incidence and severity of obesity and metabolic complications are constantly rising, and drug therapy has the potential to cause adverse effects (Sonnenburg and Bäckhed, 2016; Dahiya et al., 2017). The necessity for alternative therapy is increasing, and attention has been drawn by the effectiveness and utilization of probiotic lactobacilli (Aryana and Olson, 2017). Animal studies and clinical studies have reported the anti-obesity effects of probiotic lactic acid bacteria and improvements in metabolic disorders (Dahiya et al., 2017). In this study, the administration of *L. sakei* AMD14 suppressed weight gain and reduced body fat mass in a high-fat mouse model as shown in our previous study (Won et al., 2020b). Although, there is no significant difference in total caloric consumption, the difference in physical indicators is confirmed to be an inhibitory effect by *L. sakei* ADM14 (Figure 1). The reduction in the weight of subcutaneous fat tissue, as well as reduction in the weight of the epididymal fat tissue also observed previous study (Won et al., 2020b), appears to induce the overall decrease in lipid accumulation in mice administered with *L. sakei* AMD14. In this study, it was found that the weight of the liver was decreased, and as a result of microscopic observation through Oil Red O staining of the liver tissue, a decrease in the size of the adipocytes was observed (Figures 1D,E). This is seen as evidence that the expansion of adipocytes and accumulation of lipids in the liver were inhibited. Cardiovascular disease and lipidemia are major metabolic complications that occur and intensify because of obesity (Cani and Delzenne, 2009). These metabolic disorders are mainly caused by an increase in blood cholesterol and TGs (Yoo and Kim, 2016). The significant increases in total cholesterol, TG, and glucose in serum of the HD group were consistent with the predicted characteristics of obesity. Administration of *L. sakei* ADM14 suppressed and improved these indicators and confirmed the possibility of preventing symptoms related to lipidemia. The improvement in blood lipids after administration of *L. sakei* ADM14 could be evaluated on the basis of another case that the probiotic lactic acid bacteria effectively improved the serum markers of high-fat diet mice (Wang et al., 2012).

To confirm the potential mechanism by which *L. sakei* ADM14 inhibits obesity in a high-fat diet mouse model, genetic expression analysis of epididymal adipose tissue was performed (Figure 2). Adipose tissue is one of the important organs that regulate lipid metabolism (Schneeberger et al., 2015). Adipocyte transcription factors *Pparγ* and *C/EBPα* play an important role in the development of adipocytes (Rosen et al., 1999; Jeong et al., 2008), and our results showed a tendency to decrease in the expression of both genes. It has been reported that these transcription factors have a positive correlation with lipid accumulation rates (Park et al., 2009). Additionally, the decreased expression of *Srebp1C*, which is involved in fatty acid synthesis, and *Fas*, which is involved in regulating lipid production, suggests that the accumulation of lipids in adipocytes was inhibited. Likewise, *aP2*, which is related to adipose production, and *CD36* and *Lpl*, which are related to lipid storage showed decreased expression and through this, it could be confirmed that a change occurred in adipose tissue metabolism. The expression of *Pparγ*, *C/EBPα*, *Fas*, *aP2*, and *CD36* in epididymal fat was confirmed to decrease also in this study as shown previously (Won et al., 2020b). From these results, it could be confirmed that administration of *L. sakei* ADM14 may cause changes in adipose tissue metabolism by affecting transcription factors, lipid production, or lipid storage. Low-grade chronic inflammation of adipose tissue is one of the major factors in obesity-induced insulin resistance and metabolic disorders (Gregor and Hotamisligil, 2011). As the expression of inflammation-related genes in adipose tissue was decreased, it was also confirmed that low-grade inflammation was alleviated (Figure 2; Won et al., 2020b).

Nonalcoholic fatty liver disease is representative of the symptoms induced by obesity (Canfora et al., 2019). The balance of lipid metabolism in the liver is disrupted, causing excessive lipogenesis and hepatic lipid accumulation (Rotman and Sanyal, 2017; Friedman et al., 2018). NAFLD can lead to liver damage and develop into fatty hepatitis, cirrhosis, or hepatocellular carcinoma (Le Roy et al., 2013). Fundamentally, the increase in the expression of major genes related to lipogenesis in liver tissue seems to be one of the biggest causes. Glucose absorbed in the intestine regulates the transcription of lipogenic genes through *ChREBP* (Kawaguchi et al., 2001). In our results, it appears that the decreased expression of *ChREBPα/β* resulted in the decreased expression of adipogenic genes, thus affecting the reduction of hepatic lipid accumulation in the liver. The expression of fatty acid synthesis-related genes such as *Pparγ*, *Fas*, and *Acc1* decreased with the inhibition of glucose catabolism. The expression of *Srebp1C* targeting *Fas* and *Acc1* also decreased. The main characteristic of NAFLD is the accumulation of TG in the liver (Eslamparast et al., 2013). In a state of excess energy such as obesity, the activity of *Dgat*, which catalyzes TG synthesis, increases (Choi and Diehl, 2008). The increase in free fatty acid delivered to the liver also upregulates the synthesis of TG and ultimately increases the likelihood of developing NAFLD (Choi and Diehl, 2008). The results of our study showed that the expression of *Dgat1* and *Dgat2* was reduced in the final stage of TG synthesis. Likewise, a reduction in the uptake of free fatty acid through the reduction of *Lpl* and *CD36* expression seems to affect the process of TG synthesis. In our results, it was confirmed that the expression of *Dgat1* and *Dgat2*, which catalyzes TG synthesis, was decreased. Likewise, it appears that a reduction in the uptake of free fatty acid to the liver through a decrease in *Lpl* and *CD36* expression influenced the TG synthesis. β-oxidation is one of the key pathways for fatty acid metabolism in the liver (Lu et al., 2014). *Pparα* and *Cpt1α* were upregulated in the HDA group compared to the HD group. This has been shown to upregulate lipolysis and fatty acid oxidation. In this study, although NAFLD is shown not to be caused by high-fat diet, genetic analysis in the mechanisms of lipid metabolism in the liver suggests that *L. sakei* ADM14 downregulates lipid production and upregulates fatty acid oxidation-related genes, while regulating hepatocytes lipid accumulation and a reduction in TG synthesis (Figure 3).

A high-fat diet can contribute to changes in the gut microbiome and can lead to metabolic imbalances caused by gut microbiota dysbiosis (Wang et al., 2011; Wu et al., 2017). In addition, gut microbiota dysbiosis has recently been reported to be associated with liver diseases as well as metabolic diseases such as obesity (Da Silva et al., 2018; Kong et al., 2018). Therefore, for the prevention and treatment of metabolic diseases, an analysis targeting the gut microbiota must be conducted (Li et al., 2020). As a result of analyzing the fecal microbiota, we observed a significant decrease (*p*<0.05) in the *Firmicutes* to *Bacteroidetes* ratio in the HDA group compared to the HD group at the phylum level as also shown in cecal microbiota from our previous study (Won et al., 2020b; Figure 2). The main cause was the difference in the relative abundance of the *Bacteroidetes* between HDA and HD groups over 10weeks. In particular, relative abundance of the *Bacteroidetes* after 10weeks from the HDA group was also similar with that of cecum shown in the study of Won et al. (2020b). In the analyses of the fecal microbiota at three different times (1, 6, and 10weeks), the increase in *Firmicutes* ratios from 1 to 6weeks was much greater than that from 6 to 10weeks in both the HD and HDA groups, whereas the decrease in *Bacteroidetes* ratios was greater from 6 to 10weeks in the HD group but was greater from 1 to 6weeks in the HDA group (Figure 4A). The *Proteobacteria* ratios from 6 to 10weeks were decreased in the HDA group unlike in the HD group (Figure 4A). The administration of *L. sakei* ADM14 from 1 to 10weeks does not affect *Firmicutes* ratios between the HD and HDA groups but affect the ratios of *Bacteroidetes* and *Proteobacteria* in the HDA group. In the study of Won et al. (2020b), administration of *L. sakei* ADM14 was shown to increase *Firmicutes* ratios in the cecal microbiota. These results suggest that the failure to increase the *Firmicutes* ratio in fecal microbiota despite the administration of *L. sakei* might be due to colonization of *L. sakei* ADM14 in the intestine. The *Firmicutes* can produce more harvestable energy than *Bacteroidetes*, and when the relative abundance of *Firmicutes* increases and *Bacteroidetes* decreases, the absorption of calories in the digestive tract increases (Turnbaugh et al., 2006; Komaroff, 2017). This difference in terms of energy harvesting promotes metabolic diseases such as obesity. In our results, the administration of *L. sakei* ADM14 induces a change in the proportion of the dominant taxa at the phylum level and suggests that differences in energy resources may occur. At the genus level, *Bacteroides*, *Oscillibacter*, and *Alistipes*, which have higher proportions in the HDA group compared to the HD group, were reported to have a negative correlation with obesity (Dahiya et al., 2017; Thingholm et al., 2019). *Bacteroides* and *Alistipes* were also observed to have higher proportions in cecal sample of the HDA group in previous study by Won et al. (2020b). *Bacteroides* and *Alistipes* are known to be the major producers of short chain fatty acids such as acetic acid and butyric acid (Borton et al., 2017; Yin et al., 2018). In fecal microbiota, members of genus *Alistipes*, family *Muribaculaceae* and species *Bacteroides acidifaciens* were notable bacteria whose relative abundance was significantly changed by the administration of *L. sakei* ADM14 (Figure 4E). The genus *Alistipes* is found at low levels in the intestines of patients with hepatocellular carcinoma, colitis, and NAFLD and has been reported to have protective effects against some diseases including liver fibrosis, colitis, and cardiovascular disease (Parker et al., 2020). *Muribaculaceae*, previously known as family S24-7, was reported as representative bacteria with low abundance in mice fed a high-fat diet (Lagkouvardos et al., 2016). *Bacteroides acidifaciens* has been reported to be effective in preventing metabolic disorders such as obesity by controlling the host lipid metabolism (Yang et al., 2017). Overall, *L. sakei* ADM14 changed the composition of gut microbiota, suggesting the possibility of preventing metabolic disorders caused by dysbiosis. In particular, an increase in the abundance of *Alistipes* through gut microbiota modification helps to prevent diseases such as fatty liver through gut-liver axis modulation.

## Conclusion

In this study, *Latilactobacillus sakei* ADM14 was found to inhibit adipogenesis by regulating the expression level of lipid metabolism in adipose tissue and liver tissue in a high-fat diet mouse model. In addition, fecal microbial community analysis suggested that the composition and ratio of gut microbiota were modified to induce changes in energy harvest, and the possibility of preventing metabolic disorders through modulation of the gut-liver axis. To our knowledge, there are few studies to evaluate the relationships among obesity, lipid metabolism in both epididymal fat and liver, and fecal microbiomes at different times by probiotic strain, particularly lactic acid bacteria isolated from kimchi. Further research and clinical studies are needed to evaluate their applicability and effectiveness in human.

## Data Availability Statement

The datasets presented in this study can be found in online repositories. The names of the repository/repositories and accession number(s) can be found at: https://www.ncbi.nlm.nih.gov/ (PRJNA751416, PRJNA751419, PRJNA751420, PRJNA751421, PRJNA751431, and PRJNA751433).

## Ethics Statement

The animal study was reviewed and approved by Animal Care and Use Committee of the College of Biotechnology at Sungkyunkwan University (approval number: SKKUIACUC-18-04-14-3).

## Author Contributions

S-MW and MS performed the experiments of this study and reviewed the manuscript. MK supported some experiments of this study. KP discussed the results of this study. J-HY designed this study and wrote manuscript. All authors contributed to the article and approved the submitted version.

## Funding

This work was supported by the “Cooperative Research Program for Agriculture Science and Technology Development (project no. PJ015247)” of the Rural Development Administration, Republic of Korea and a Research Initiative Program of Sungkyunkwan University, Republic of Korea.

## Conflict of Interest

The authors declare that the research was conducted in the absence of any commercial or financial relationships that could be construed as a potential conflict of interest.

## Publisher’s Note

All claims expressed in this article are solely those of the authors and do not necessarily represent those of their affiliated organizations, or those of the publisher, the editors and the reviewers. Any product that may be evaluated in this article, or claim that may be made by its manufacturer, is not guaranteed or endorsed by the publisher.
