# Supplementary material for: Oral Administration of Latilactobacillus sakei ADM14 Improves Lipid Metabolism and Fecal Microbiota Profile Associated With Metabolic Dysfunction in a High-Fat Diet Mouse Model
Source: Front Microbiol. 2021 Oct 6;12:746601. doi: 10.3389/fmicb.2021.746601 (PMC8527011; doi:10.3389/fmicb.2021.746601)
Supplement: Supplementary file 1 [file Data_Sheet_1.PDF]

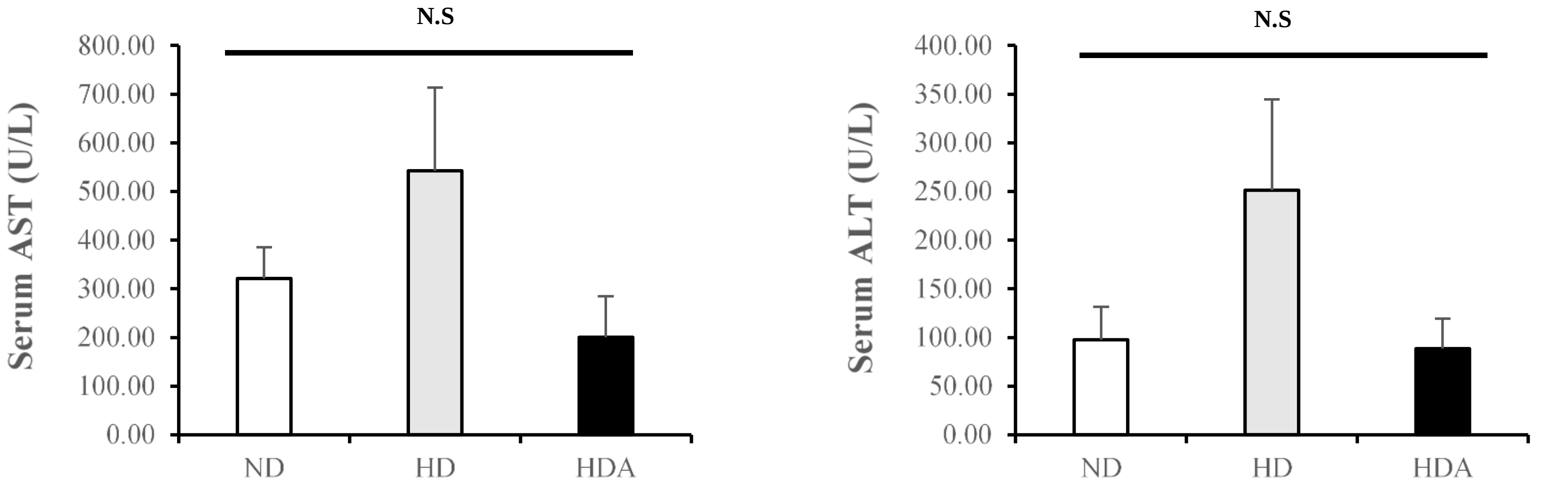

SUPPLEMENTARY FIGURE S1 Effect of administered *L. sakei* ADM14 on the toxicity biomarkers. (A) Serum AST. (B) Serum ALT. Values are shown as mean ± SEM (n = 5).

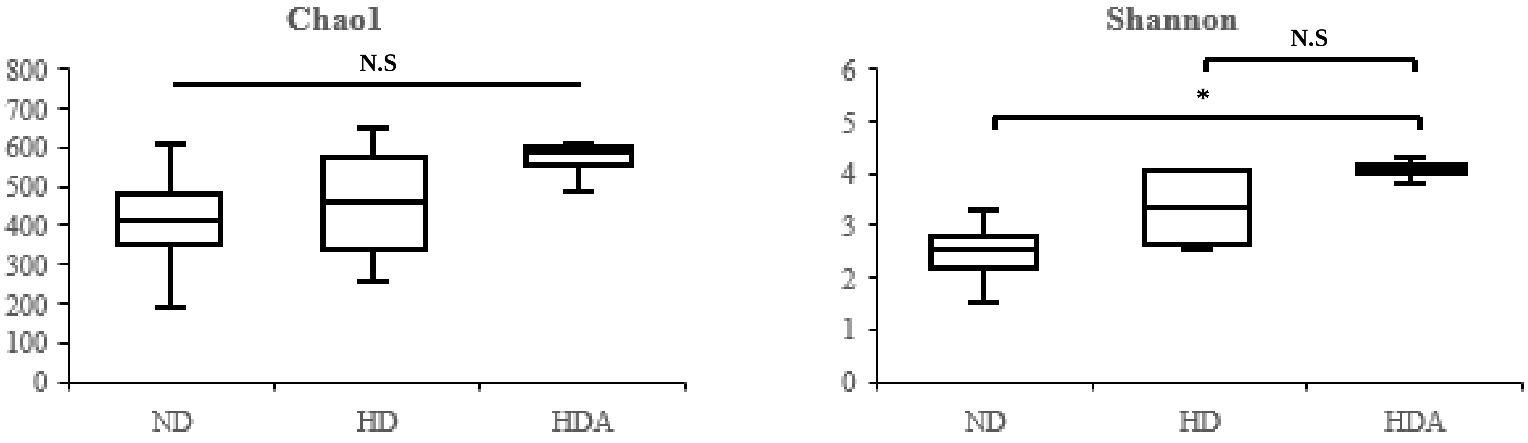

SUPPLEMENTARY FIGURE S2 Alpha diversity indexes between groups. (A) The Chao1 richness estimator. (B) Shannon's diversity index. The nonparametric Wilcoxon signed rank test for paired data. Results are shown as mean ± SEM (n = 4-6). Significant differences between groups are indicated as *p<0.05.

SUPPLEMENTARY TABLE S1 Composition of high-fat and normal-fat diets used in this study.

**Ingredient (g)***	**High-fat diet (60% kcal% fat)**	**Normal-fat diet (10% kcal% fat)**
Protein (kcal%)	20	20
Carbohydrate (kcal%)	20	70
Fat (kcal%)	60	10
Casein, 80 Mesh	200	200
L-Cystine	3	3
Corn starch	0	315
Maltodextrin 10	12.5	35
Sucrose	68.8	350
Cellulose, BW200	50	50
Soybean oil	25	25
Lard‡‡‡‡	245	20
Mineral mix, S10026	10	10
Dicalcium phosphate	13	13
Calcium carbonate	5.5	2.2
Potassium citrate 1 H_2O	16.5	16.5
Vitamin mix, V1001	10	10
Choline bitartrate	2	2
FD&C dye	0.05 (Blue dye #1)	0.05 (Yellow dye #5)

*Formulated by E. A. Ulman, Ph.D., Research Diets, Inc., 8/26/98 and 3/11/99.

†Typical analysis of cholesterol in lard = 0.72 mg/gram. Cholesterol (mg)/4057 kcal = 14.4, Cholesterol (mg)/kg = 13.6

SUPPLEMENTARY TABLE S2 Primer sequences for genes related to lipid metabolism and inflammation.

Gene	Forward primer sequence (5' - 3')	Reverse primer sequence (5' – 3')
Pparγ	CCAGAGCATGGTGCCTTCGC	CAGCAACCATTGGGTCAGCTC
Pparα	ATGCCAGTACTGCCGTTTTC	CCGAATCTTTCAGGTCGTGT
C/EBPα	GAACAGCAACGAGTACCGGGTA	GCCATGGCCTTGACCAAGGAG
Srebp1C	GGT TTT GAA CGA CAT CGA AGA	CGG GAA GTC ACT GTC TTG GT
Fas	GCTGCTGTTGGAAGTCAGC	AGTGTTCGTTCCTCGGAGTG
aP2	CACCGCAGACGACAGGAAG	GCACCTGCACCAGGGC
Lpl	TGGAGAAGCCATCCGTGTG	TCATGCGAGCACTTCACCAG
CD36	GGCCAAGCTATTGCGACAT	CAGATCCGAACACAGCGTAGA
ChREBPα	CGACACTCACCCACCTCTTC	TTGTTCAGCCGGATCTTGTC
ChREBPβ	TCTGCAGATCGCGTGGAG	CTTGTCCCGGCATAGCAAC
Cpt1α	GATGTGGACCTGCATTCCTT	TCTTGTAATGTGCGAGCTG
Acc1	ACAGTGGAGCTAGAATTGGAC	ACTTCCCGACCAAGGACTTTG
Dgat1	GCTGATCCAACAGTGGATGG	GACGCTCAATGATCCGTGAATA
Dgat2	GAGGGGTCTGGGCGATGGGGCACT	CGACGGTGGTGATGGGCTTGGAGT
TNFα	GCCACCACGCTCTTCTGCCT	GGCTGATGGTGTGGGTGAGG
MCP-1	TCTGGACCCATTCCTTCTTG	AGGTCCCTGTCATGCTTCTG
IL-6	CAAGAAAGACAAAGCCAGAGTCCTT	TGGATGGTCTTGGTCCTTAGCC
36B4	AGATGCAGCAGATCCGCAT	GTTCTTGCCCATCAGCACC

SUPPLEMENTARY TABLE S3 Primer sequences for V3 to V4 regions of the 16S rRNA gene.

Primer	Sequence (5' – 3')
341F	TCGTCGGCAGCGTCAGATGTGTATAAGAGACAGCCTACGGGNGGCWGCAG
805R	GTCTCGTGGGCTCGGAGATGTGTATAAGAGACAGGACTACHVGGGTATCTAATCC
